# Acetic Acid Detection Threshold in Synthetic Wine Samples of a Portable Electronic Nose

**DOI:** 10.3390/s130100208

**Published:** 2012-12-24

**Authors:** Miguel Macías Macías, Antonio García Manso, Carlos Javier García Orellana, Horacio Manuel González Velasco, Ramón Gallardo Caballero, Juan Carlos Peguero Chamizo

**Affiliations:** 1 University Center of Merida, University of Extremadura, Sta. Teresa de Jornet, 38, Mérida 06800, Spain; E-Mail: jcpeg@unex.es; 2 Polytechnic School, University of Extremadura, Cáceres 10003, Spain; E-Mails: antonio@nernet.unex.es (A.G.M.); horacio@nernet.unex.es (H.M.G.V.); ramon@nernet.unex.es (R.G.C.); 3 Faculty of Science, University of Extremadura, Avda. Elvas s/n, Badajoz 06006, Spain; E-Mail: carlos@nernet.unex.es

**Keywords:** electronic nose, neural network, principal component analysis

## Abstract

Wine quality is related to its intrinsic visual, taste, or aroma characteristics and is reflected in the price paid for that wine. One of the most important wine faults is the excessive concentration of acetic acid which can cause a wine to take on vinegar aromas and reduce its varietal character. Thereby it is very important for the wine industry to have methods, like electronic noses, for real-time monitoring the excessive concentration of acetic acid in wines. However, aroma characterization of alcoholic beverages with sensor array electronic noses is a difficult challenge due to the masking effect of ethanol. In this work, in order to detect the presence of acetic acid in synthetic wine samples (aqueous ethanol solution at 10% v/v) we use a detection unit which consists of a commercial electronic nose and a HSS32 auto sampler, in combination with a neural network classifier (MLP). To find the characteristic vector representative of the sample that we want to classify, first we select the sensors, and the section of the sensors response curves, where the probability of detecting the presence of acetic acid will be higher, and then we apply Principal Component Analysis (PCA) such that each sensor response curve is represented by the coefficients of its first principal components. Results show that the PEN3 electronic nose is able to detect and discriminate wine samples doped with acetic acid in concentrations equal or greater than 2 g/L.

## 1. Introduction

Poor winemaking practices and bad storage conditions usually lead to wine spoilage, which can result in an unpleasant characteristic of a wine, known as a wine fault or wine defect. Among the causes of this wine defects we could cite, for instance, excessive or insufficient exposure of the wine to oxygen or to sulphur, bad hygiene conditions at the winery, overextended maceration of the wine, faulty fining, filtering and stabilization of the wine, the use of dirty oak barrels, over extended barrel aging or the use of poor quality corks. Besides, there are other factors outside the winery that can contribute to the wine spoilage, for example bad storage conditions, or exposure to excessive temperature fluctuations [1].

There are a lot of compounds that can cause wine defects, most of which are naturally present in the wine itself, but at insufficient concentrations to be considered as an unpleasant characteristic. In fact, these concentrations may be responsible for the positive characters of a wine. However, the excessive concentration of these compounds can obscure the flavours and aromas that the wine should be expressing, reducing the quality of the wine and making it less appealing, even sometimes undrinkable [2].

One of the most common wine faults is the excessive volatile acidity (VA), typically acetic acid. Acetic acid in wine can be contributed by many wine spoilage yeasts and bacteria and can cause a wine to take on aromas of vinegar, salad dressing, ketchup and barbeque sauce while reducing varietal character. VA is detectable at the 0.6–0.9 g/L level and concentrations greater than 1.2–1.3 g/L can result unpleasant. Therefore, it is very important for the wine industry to find a fast analytical method, like an electronic nose, for real-time monitoring of the concentration of acetic acid in wines. However, the analysis of foodstuffs with semiconductor-based electronic noses is known to be a difficult challenge due to the non specificity of the sensor arrays and principally to the presence of high ethanol and water concentrations in the samples. Indeed, in a wine sample, the aroma compounds amount only to about 1 g/L, while water and ethanol amount to about 900 and 100 g/L, respectively [3]. Water contributes to the shortening of the sensor span life and increases the signal drift with time, while ethanol masks the presence of other volatile compounds [4].

Although alcoholic beverage discrimination using electronic noses has been already reported in the scientific literature, it is believed that this discrimination most often reflects mere variations in the sample alcohol content and not true differences in the aroma profiles [5–8]. For this reason, in this work we use synthetic matrices with fixed alcohol content. Specifically, we intend to study the acetic acid detection threshold of the PEN3 electronic nose in synthetic wine samples [aqueous ethanol solution at 10% (v/v)].

In other works, authors have argued that electronic noses in many real applications may encounter bulk compounds that, only owing to their high concentrations, interfere with the detection of target solutes, as is the case for water (humidity) and ethanol in foodstuffs, and propose ingenious solutions for the requirements of each specific application. Examples of these techniques can be found in [9] where the authors used Solid Phase Micro Extraction in combination with an electronic nose, in [10] where the volatiles were desorbed from a Tenax TA, and in [11] where a heated preconcentration tube was used as a dispersive element for a QCM array. In [12], back-flush gas chromatography was used to remove water and ethanol from the other volatiles, while pervoration was suggested as a sample pretreatment in [13], and finally a chromatograph with an SAW sensor as the detector was used in the zNose “Electronic nose” [14].

These arrangements make fuzzier the border between classical analytical systems, electronic nose technology, and detectors for specific substance classes, or even single compounds. Though sensor-based improvements of the selectivity are obtained with those systems, they all have the disadvantage of an increase in setup complexity, in analysis time and in cost, which are the real motivations of the electronic noses.

The main goal of this work is to test the viability of the use of a basic electronic nose (a sensor array without special arrangements) to detect excessive concentrations of acetic acid in synthetic wine samples. In Section 2 we describe the methodology, the electronic nose, and the characteristics of the autosampler used to take the measurements. Besides, a brief description of how principal component analysis and neural networks are used has been included. In Section 3 we present the results produced by our system when tested using aqueous ethanol solutions without acetic acid and with acetic acid in different concentrations. Finally, in Section 4 the conclusions of the present work are shown.

## 2. Methodology

**Figure 1.**
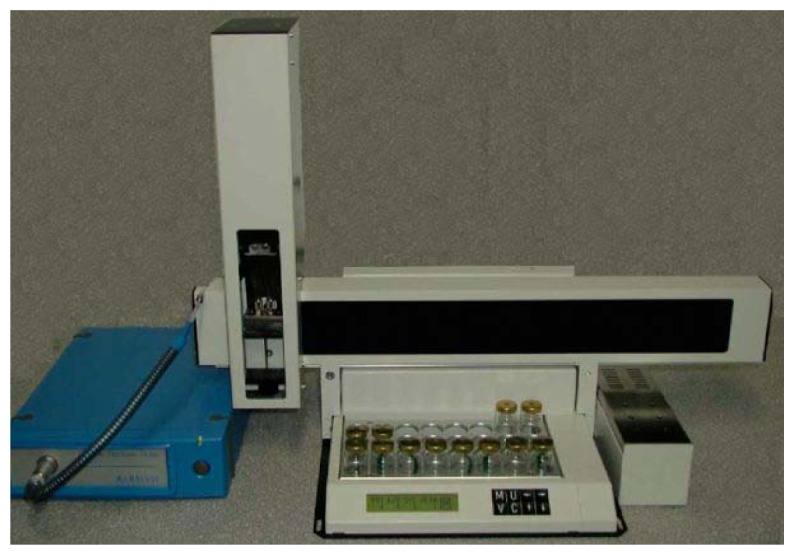
Airsense HSS32 autosampler connected to the portable electronic nose PEN3.

In this work we have used a Portable electronic nose (PEN3) in combination with a Headspace Autosampler HSS32 (Figure 1), both made by Win Muster Airsense (WMA) Analytics Inc. (Schwerin, Germany). This system has been used for different tasks, for example to characterize peach cultivars and to monitor their ripening stage [15], to analyze volatile emissions from wastewater [16] and to monitor storage shelf life of tomatoes [17].

The portable electronic nose (PEN) consists of a sampling apparatus, a detector unit containing the array of sensors, and a pattern recognition software (Win Muster v.1.6) for data recording and elaboration. The sensor array is composed of 10 metal oxide semiconductor (MOS) type chemical sensors whose characteristics can be observed in Table 1. The sensor response is expressed as resistivity (Ohm) and relies on changes in conductivity induced by the adsorption of molecules in the gas phase, and on subsequent surface reactions.

Measurements are taken using the dynamic headspace technique [18]. In dynamic headspace sampling, the headspace of the vials is continuously swept into the detector by a clean purge flow for analysis. This way, the gaseous analyte concentration immediately above the liquid phase is kept as low as possible to increase the evaporation rate. This evaporation rate depends on the surface area, the analyte surface concentration, the analyte volatility and the sample temperature.

**Figure 2.**
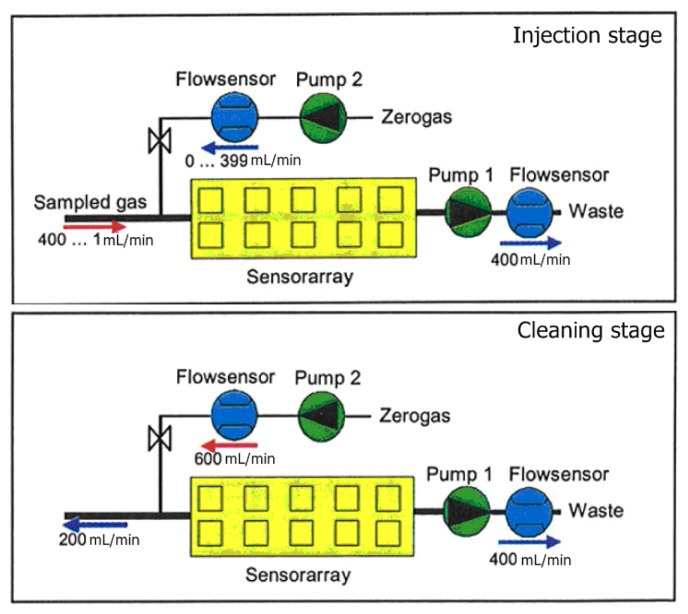
Schematic diagrams of the gas flow of PEN3 during the electronic nose measurements.

The measurement phase of the electronic nose is divided into two stages, injection and cleaning. In the injection phase the volatiles of the sample are transported to the sensor chamber to be analyzed by the array of sensors, and in the cleaning phase all traces of volatiles must be removed of the electronic nose to avoid interference with the next measurement. In Figure 2, we can observe the gas flow in each stage. In the injection phase, pump 1 sucks the sample gas compounds through the sensor array and pump 2 transfers filtered reference air into the sensor array. This arrangement allows dilution and avoids saturation of the sensors due to, for example, high concentrations of ethanol. In the cleaning phase, the cleaning air flow of pump 2 is used to rinse the system. Due to the higher flow rate of pump 2, the original gas flow direction at the inlet is inverted.

The Airsense HSS32 headspace sampler is a dedicated sampling unit specially modified by Airsense in order to be used with the dynamic headspace technique. The sampler provides automatic analysis of up to 32 samples within the temperature range of ambient to T = 180 °C. Samples are conditioned inside sealed 10 mL glass vials. After that, the vials are thermostated, the injection needles are driven through the septum into the vial and, using the e-nose as detector, take a sample from the headspace into the sensor array. The whole system is controlled by the WinMuster software.

There are a lot of parameters that have to be adjusted in order to find a suitable response of the electronic nose. The most important ones are the injection time, the flush or cleaning time, the injection flows of the sampled gas, the zerogas and the waste gas for the PEN3, and the temperature of the vials for the autosampler.

After numerous tests, measurements were taken with the following values of the adjustable parameters of the PEN electronic nose and the auto sampler:

- Injection sampled gas: 20 mL/min.
- Zerogas flow: 380 mL/min.
- Waste flow: 400 mL/min.
- Injection time: 3 s.
- Cleaning time: 20 min.
- Vials temperature: 40 °C.

As we showed above the electronic nose contains 10 sensors, but due to “the curse of the dimensionality”, a 10-dimensional characteristic vector representative of the samples can be very large for the dimensions of our prototype set. Therefore, we need to reduce the dimensionality of the initial characteristic vector. One possible way could be to apply techniques of feature selection or extraction, such as principal component analysis, independent component analysis or genetic algorithms. However, in this work, we decided to use *a priori* knowledge of the problem at issue by previously determining the sensors that better responded to the presence of acetic acid. For this task, we used synthetic wine samples doped with a very high concentration of acetic acid, and analyzed the response curve of the sensors, particularly the sections where the response to acetic acid was more significant.

**Figure 3.**
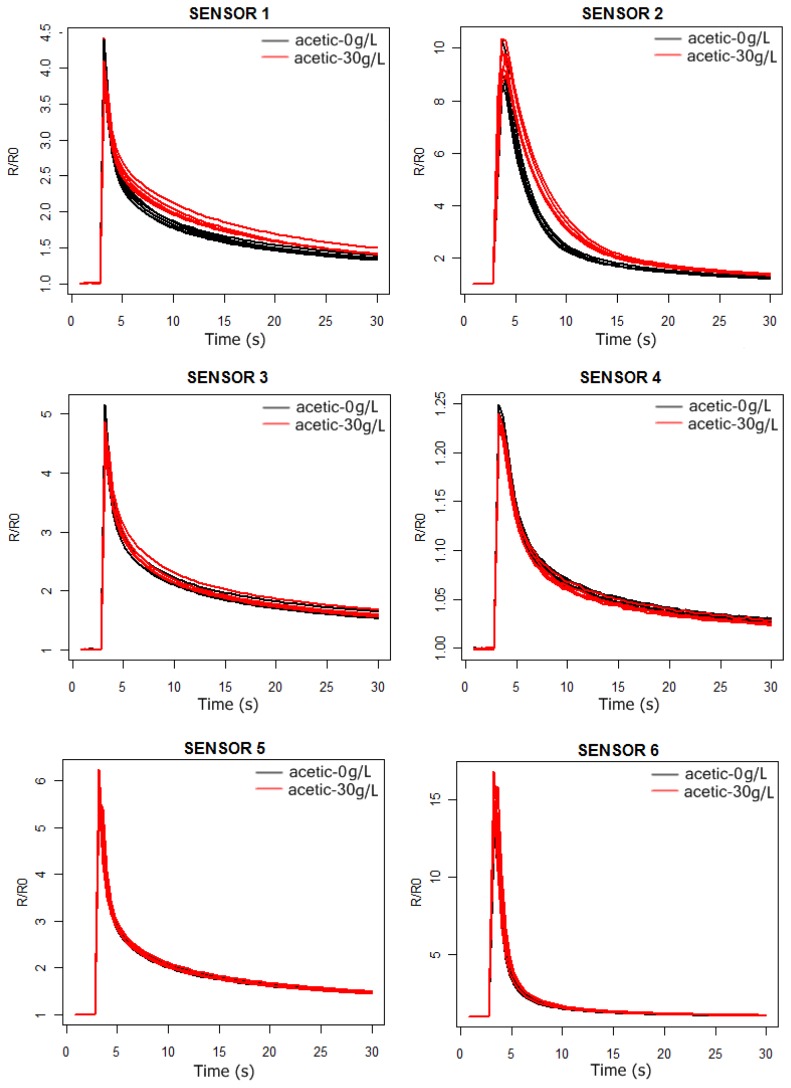
Sensor 1–6 response curves of the PEN3 electronic nose. Black: 10% aqueous ethanol solution. Red: 10% aqueous ethanol solution doped with acetic acid 30 g/L.

**Figure 4.**
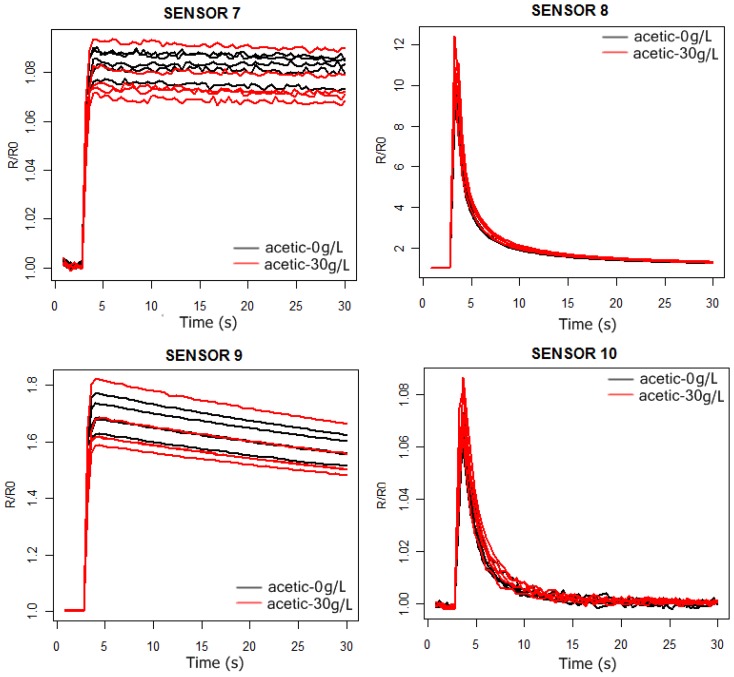
Sensor 7–10 response curves of the PEN3 electronic nose. Black: 10% aqueous ethanol solution. Red: 10% aqueous ethanol solution doped with acetic acid 30 g/L.

In Figures 3 and 4 we can observe the response curves of the PEN3 sensors for the synthetic wine samples, with and without acetic acid. In the acetic acid case the concentration is 30 g/L, much higher than the actual concentrations (around 1 g/L). In this way, we intend to check by visual inspection of the sensors response curves which ones are the best to detect the presence of acetic acid in the solutions.

**Figure 5.**
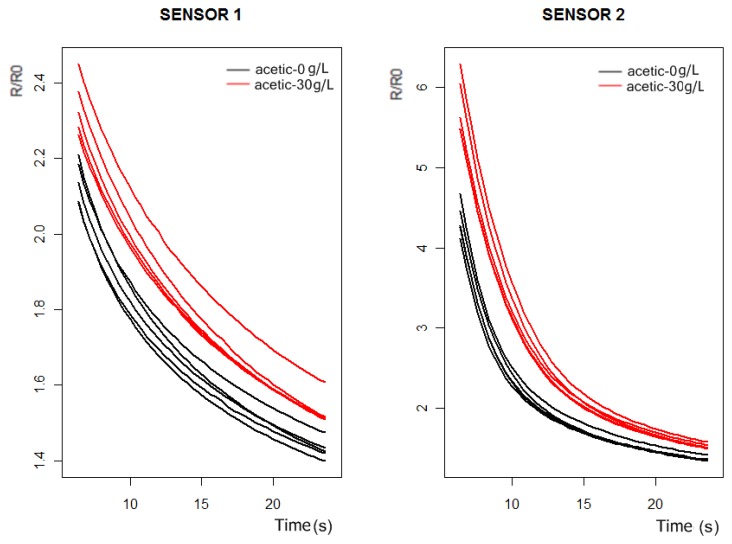
Response curves of the sensors 1 and 2 in the interval of 6 to 24 s.

As can be observed in Figures 3 and 4, the probability of detecting the presence of acetic acid will be higher by using sensors 1 and 2. Furthermore, from Figure 5 we conclude that the more significant differences in the sensors responses happen between 6 and 24 s.

With this information, now the objective is to detect the presence of acetic in much lower concentrations (actual cases) where the differences of the sensor responses are no so clear. To achieve that, we firstly characterized the sensor curve using principal component analysis (PCA) [19], and later we used a multilayer perceptron (MLP) neural network to classify the responses of the sensor, trying to determine the presence and the concentration of acetic acid.

### 2.1. Principal Component Analysis

During the measurement time of the electronic nose, the sampling period is 0.4 s, so each response curve at the interval of interest (6–24 s) is composed of 44 points. A lot of features have been proposed for the characterization of response curves of sensors: maximum, minimum, slope, average, *etc*. In this work, we propose the use of principal component analysis to reduce the dimensionality of the 44-dimensional vector that initially characterizes the sensor response.

**Figure 6.**
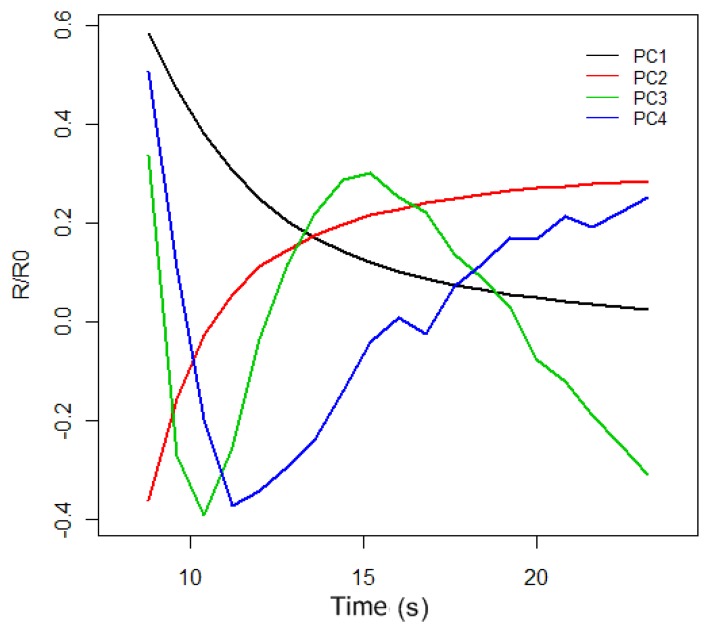
First four principal components of the PCA.

The original purpose of PCA was to reduce a large number of variables to a much smaller number of principal components (PCs), whilst retaining as much as possible the variation in the original variables. To do PCA of the response curves of Figure 5, we took 50 measurements, 25 for the synthetic wine samples doped with 30 g/L of acetic acid and 25 for the synthetic wine samples without acetic acid. If we only consider the sensor 1 and sensor 2 response curves at the interval of 6 to 24 s, we have a data matrix of 100 rows, (corresponding to 50 measurements of the sensor 1 and sensor 2), and 44 columns. Doing PCA of the data matrix we obtained the principal components (the autovectors of the covariance matrix). The first four principal components can be observed in Figure 6, and according to PCA, each initial response curve of Figure 5 can be decomposed as a linear combination of these autovectors or basis functions. The coefficients of the linear combination form the characteristic vector representative of each response curve.

In Table 2, we can observe the proportion of the variance of the first four principal components. In this case, only the first PC accumulates 96.5% of the initial variance, and therefore we only used this component to characterize each response curve. This allows us, hereinafter, to represent each electronic nose measure by a two-dimensional characteristic vector that is composed of the projections of sensor1 and sensor2 response curves onto the first principal component.

### 2.2. Neural Network Usage

To resolve the classification problem we used a classical one hidden layer perceptron trained with the package Amore of the free software environment for statistical computing and graphics R (The R Project for Statistical Computing) [20].

In order to assess the performance of our neural networks models in practice, we used k-fold cross-validation method [21]. One round of k-fold cross-validation involves partitioning our sample of data into k complementary subsets, from which k-1 subsamples are used to fit our predictive model, and the other subsample (the test set) is used to test its performance. Besides, to achieve a good generalization capability, the set used for adjusting the neural networks is divided into a training set and a validation set. The training set is used to train the neural networks models and the validation set is used to stop training when a minimum of the error over this set is reached. For each experiment we used one and two hidden layers perceptrons where the number of neurons of the hidden layer varied from 20 to 50 neurons in steps of 5 in the one-hidden-layer case and from 5 to 20 in steps of 5 in the two-hidden-layers case. To avoid local minimum each training process was repeated 10 times randomizing the initial weights. Finally, from all this experiments, the neural network model with the minimum error over the validation set was returned and its performance was measured calculating the error over the test set.

Finally, to reduce variability, multiple rounds of cross-validation were performed using different partitions, and the test results were averaged over the rounds.

## 3. Results

To find the acetic acid detection threshold, we prepared five synthetic wine samples and we doped four of them with acetic acid in different concentrations: 1, 2, 3 and 4 g/L respectively. Then we took ten measurements for each solution with our auto sampler and PEN3 electronic nose.

Moreover, given that the big problems working with electronic noses are reproducibility and repeatability, measures were taken in different days and, especially, prototypes of the different classes were taken alternatively. If we don't take the measurements in this way, the separability of classes could be due to sensors drift or instability or other causes, different from the concentration of acetic acid.

Finally, we had 50 prototypes and five classes and, as we concluded in the previous sections, each prototype is characterized by a two dimensional vector composed of the projections of sensors 1 and 2 responses onto the first PC. So, in this case, we used one and two hidden layers neural networks with two input neurons and five output neurons.

**Figure 7.**
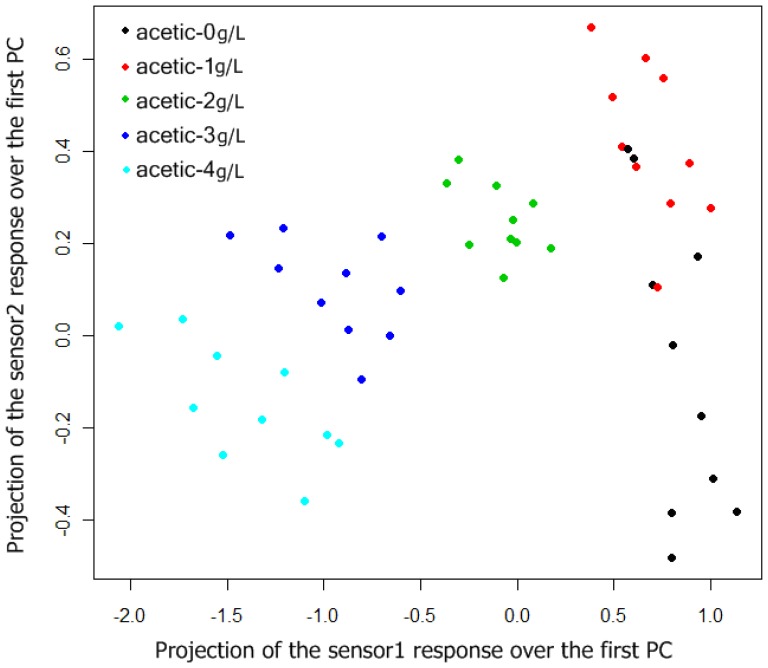
Two dimensional representation of the classification problem.

In Figure 7, a two-dimensional plot of the 50 prototypes can be observed. From this figure we must expect some misclassifications between the classes “acetic-0 g/L” and “acetic-1 g/L” and good classification results for the other classes.

**Figure 8.**
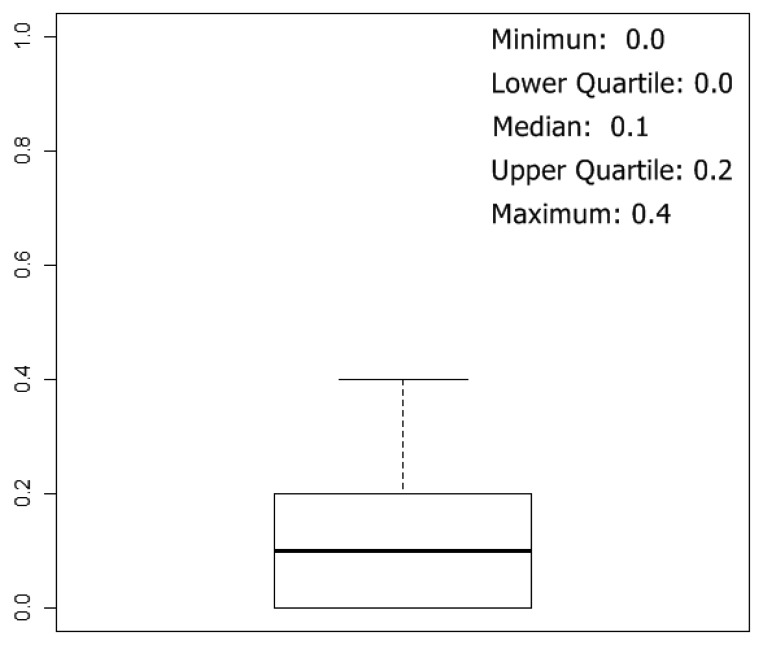
Boxplot of the error calculated over the test sets, considering the 50 simulations generated applying 10 rounds of 5-fold cross-validation over the 50 prototypes.

To estimate classification results, 5-fold cross validation method was used, and 10 rounds of cross-validation were performed, so we tested 50 neural network models. The box plot of the error over the test set of the 50 simulations can be summarized in Figure 8. The best results were obtained with networks with one hidden layer, and the mean of the number of neurons in the hidden layer, considering the 50 best networks (5 k-fold and 10 rounds of cross validation), were 29 neurons.

In Table 3, the confusion matrix of the 50 neural networks generated applying 10 rounds of 5-fold cross-validation is shown. Each network was tested over one test set of 10 prototypes, so we made 500 individual classifications. As might be expected, observing Figure 8, almost all misclassifications happen between the classes “acetic-0 g/L” and “acetic-1 g/L”. On the other hand, correct classifications for the other classes are over 96%.

**Figure 9.**
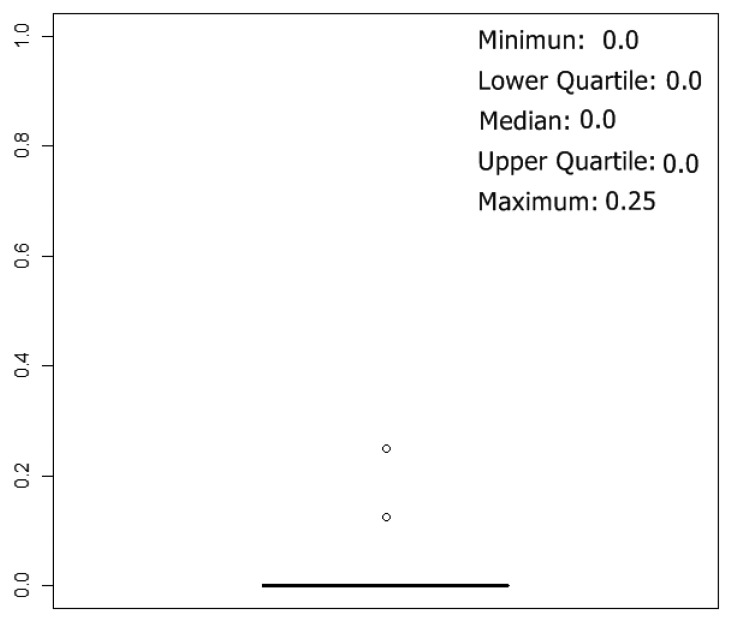
Boxplot of the error calculated over the test sets, considering the 50 simulations generated applying 10 rounds of 5-fold cross-validation over the 40 prototypes. Class Prototypes of acetic-1 g/L class were not used.

In Table 4 and Figure 9 we can observe the classification results and box plot when removing the prototypes of acetic-1 g/L class from the prototypes set. In this case, networks had two input and four output neurons, and we made 50 simulations applying 10 rounds of 5-fold cross-validation over the 40 prototypes. Each network was tested with one test set of 8 prototypes, so we made 400 individual classifications. As can be observed, correct classification results are over 98%.

## 4. Conclusions

In this paper we have proved that gas sensors, in particular sensors W1S and W5S installed in the PEN3 electronic nose, are able to detect the presence of acetic acid in 10% aqueous ethanol solutions when the concentration is equal or greater than 2 g/L. As has been shown, the acetic acid presence mainly affects the sensors response curves during the cleaning stage, in a period of time between 6 and 24 s for an injection time of 3 s. However, the maximum points in the sensors response curves, reached approximately at 5 s, are similar for solutions with and without acetic acid, so we can conclude that these maximum values only depend on the ethanol concentration in the aqueous solution.

Though different experiments have been carried out using other sensors' response curves (for instance, sensor 3–sensor 10), and also using the second PC (which accumulates 3.4% of the initial variance, as shown in Table 2), the classification results did not improve.

On the other hand, taking into account that our objective is to detect concentrations of VA considered as unpleasant (*i.e.*, greater than 1.2–1.3 g/L), and considering also that the probability of success for that concentrations (over 69%) is low for a real time monitoring application, our current work is focused on finding better values for the adjustable parameters of the PEN3 electronic nose and the autosampler, in order to improve classification results.

## Figures and Tables

**Table 1.** Description of the sensors installed in the portable electronic nose PEN3 [15].

**Number**	**Sensor name**	**General description**	**Reference**

1	W1S	Aromatic compounds	Toluene, 10 mg·kg^−1^
2	W5S	Very sensitive, broad range sensitivity, react on nitrogen oxides, very sensitive with negative signal	NO_2_, 1 mg·kg^−1^
3	W3C	Ammonia, used as sensor for aromatic compounds	Benzene, 10 mg·kg^−1^
4	W6S	Mainly hidrogen, selectively	H_2_, 100 μg·kg^−1^
5	W5C	Alkanes, aromatic compounds, less polar compounds	Propane, 1 mg·kg^−1^
6	W1S	Sensitive to methane (environment) *ca.* 10 mg·kg^−1^. Broad range, similar to n. 8	CH_3_, 100 mg·kg^−1^
7	W1W	Reacts on sulphur compounds, H_2_S 0.1 mg·kg^−1^. Otherwise sensitive to many terpenes and sulphur organic compounds, which are important for smell, limonene, pyrazine	H_2_S, 1 mg·kg^−1^
8	W2S	Detect alcohols, partially aromatic compounds, broad range	CO, 100 mg·kg^−1^
9	W2W	Aromatic compounds, sulphur organic compounds	H_2_S, 1 mg·kg^−1^
10	W3S	Reacts on high concentration >100 mg·kg^−1^, sometime very selective (methane)	CH_3_, 10 CH_3_, 100 mg·kg^−1^

**Table 2.** Importance of the first four components.

	**Standard deviation**	**Proportion of Variance**
PC1	1.34	0.965
PC2	0.253	0.034
PC3	0.018	0.00018
PC4	0.010	5.47 e-5

**Table 3.** Confusion matrix, calculated with the 50 simulations generated applying 10 rounds of 5-fold cross-validation over the 50 prototypes.

	**Acet0**	**Acet1**	**Acet2**	**Acet3**	**Acet4**
Acet0	69	31	0	0	0
Acet1	17	81	2	0	0
Acet2	2	0	98	0	0
Acet3	0	0	0	96	4
Acet4	0	0	0	0	100

**Table 4.** Confusion matrix, calculated with the 50 simulations generated applying 10 rounds of 5-fold cross-validation over the 40 prototypes.

	**Acet0**	**Acet2**	**Acet3**	**Acet4**
Acet0	99	1	0	0
Acet2	0	100	0	0
Acet3	0	2	98	0
Acet4	0	0	0	100
